# Factors Affecting Wound Healing after the Wide Surgical Excision of Hidradenitis Suppurativa Lesions

**DOI:** 10.3390/jcm13185598

**Published:** 2024-09-21

**Authors:** Anne-Cecile Ezanno, Gaëtan Texier, Joffrey Marchi, Anne-Claire Fougerousse

**Affiliations:** 1 Department of Digestive Surgery, Begin Military Teaching Hospital, 94160 Saint-Mandé, France; 2 Armed Forces Epidemiology and Public Health Center, 13014 Marseille, France; 3 VITROME, IHU-Méditerranée Infection, Aix Marseille University, 13005 Marseille, France; 4 Department of Dermatology, Begin Military Teaching Hospital, 94160 Saint-Mandé, France

**Keywords:** acne inversa, hidradenitis suppurativa, surgery, wide excision, wound healing

## Abstract

**Background**: Hidradenitis suppurativa (HS) is a chronic and inflammatory disease. Its management depends on the stage and extent of the disease. Surgery plays an important role in treatment options. This study explores the factors influencing healing after wide excision. **Methods**: This study analyzed data from patients who underwent wide excision for HS between 2016 and 2021. **Results**: A total of 160 patients (64.4% women) were included, with surgeries performed in the axillae (70), inguinal-ano-genital (73), and other locations (17, including gluteal). The mean TTWC was 74 ± 6 days, varying from 11 to 445 days. Factors negatively influencing TTWC included axillary localization (*p* < 0.001) and the presence of another inflammatory disease (*p* = 0.017). Factors positively influencing healing were smoking (<0.001) and previous or ongoing medical treatment (antibiotherapy or biologics) (*p* = 0.011). Obesity or being overweight did not impact the TTWC in multivariate analysis, although overweight was significant in univariate analysis. **Conclusions**: While smoking cessation remains important in the management of HS, it does not appear to be a prerequisite for successful surgical treatment. Conversely, patients with an inflammatory disease or those undergoing surgery for an axillary lesion exhibit slower healing and should be informed of potential healing delays before surgery.

## 1. Introduction

Hidradenitis suppurativa (HS) is a chronic inflammatory skin disease, affecting axillary, inguinal, gluteal, anogenital, and mammary regions. Although the pathogenesis is not fully understood, current theories suggest that HS is caused by follicular occlusion, an autoinflammatory process [1], and/or genetic and/or hereditary factors [2]. The prevalence of HS is unclear, with estimates ranging from 0.033% to 4.10% [3]. HS is associated with several comorbidities, such as inflammatory bowel diseases, and especially Crohn’s disease, axial spondylarthritis and metabolic syndrome [4,5]. Obesity and smoking are considered to be important risk factors [6]. Moreover, HS has been associated with reduced quality of life, working disability, depression and anxiety [7]. The main treatment goal is to improve patients’ quality of life by reducing pain, limiting flare-ups and removing chronic lesions through surgical techniques. Treating HS is complex and requires a multidisciplinary approach, including medical treatments (antibiotics and biologics), surgery, lifestyle changes (such as smoking cessation) and weight management [1].

**Figure 1:**
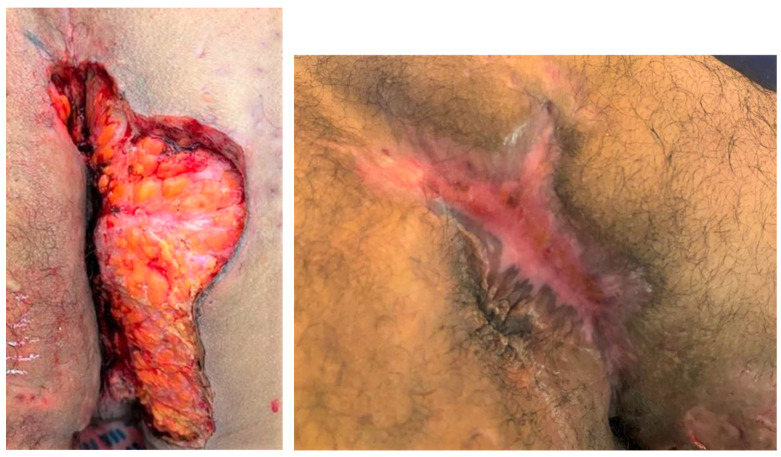
Example of surgical HS wound treated with secondary wound healing. (**Left**) Post-operative day 1, showing a buttock wound immediately after HS excision surgery. (**Right**) The same wound at 4 months after wide excision, demonstrating the healing progress achieved with secondary wound healing.

Surgery is an effective treatment option for HS, requiring wide excision to remove as much affected tissue as possible to prevent recurrence. Healing options after wide excision include primary suture, skin graft, flap reconstruction and secondary wound healing [1]. Secondary wound healing was chosen as the primary outcome of our study because it is the most frequently applied technique, as it carries a lower risk of recurrence [8] and has been demonstrated to be both functional and esthetically acceptable for patients, although it often requires a longer time to heal [9] (Figure 1). Furthermore, the strategic decision to employ secondary wound healing aligns with evidence suggesting its suitability for managing severe HS stages, which are often marked by extensive tissue involvement and a higher propensity for scarring and contractures. This approach not only addresses the immediate surgical needs, but also considers long-term outcomes, enhancing the quality of life for patients suffering from this debilitating condition [10]. By selecting secondary wound healing as our primary outcome, this study aims to elucidate the dynamics of post-surgical recovery in HS, contributing valuable insights into optimized patient care in dermatological surgery.

Many studies have addressed wound management (skin graft, flaps, etc.) and recurrence after HS surgical excision. However, few studies have focused on risk factors directly impacting the healing process following HS excision [6,9,11]. The roles of overweight, obesity and smoking—each well-documented as critical risk factors for HS—on the healing dynamics have been insufficiently studied and warrant further investigation [11,12,13,14]. This study aims to investigate the factors influencing healing time following the extensive excision of HS lesions, with a particular emphasis on the impact of overweight, smoking and lesion localization.

## 2. Materials and Methods

We conducted a retrospective cohort study, including all patients who had undergone wide excision for HS followed by secondary wound healing, between January 2018 and December 2021 at Bégin hospital (St Mandé, France). Patients were identified from medical records and our hospital information system. Exclusion criteria included patients who had undergone incisional treatment or drainage, were under 18 years old and lacked follow up at least 6 weeks after surgery. Exclusion criteria also included patients with wound sizes > 700 cm^3^, as larger wounds inherently affect healing times and could skew the results. Demographic and clinical data were extracted from medical records and our hospital information system. We collected demographic data, smoking status (current smoker vs. former smoker (interruption of more than one year) or never smoker), body mass index (BMI), history of inflammatory disease (inflammatory bowel disease (IBD), including Crohn’s disease (CD) and ulcerative colitis, or axial spondylarthritis), diabetes, cardiovascular disease, ASA score (American Society of Anesthesiologists) and HS treatment at the time of surgery (antibiotherapy in case of flare-up, background antibiotherapy or biologics). HS severity was assessed using Hurley’s classification [12]. Surgical variables included the localization of the excision, the excised area size (length, width and thickness according to pathology reports) and time to wound closure (TTWC). Time to wound closure is defined as the duration until a wound is completely closed and fully re-epithelialized. The primary endpoint was established as complete wound healing 90 days after surgery, defined by the complete re-epithelialization and cessation of daily dressings. The secondary endpoint was the time required to achieve complete wound healing, measured from the date of surgery. The choice of a 90-day period is based on established clinical observations that healing following HS surgery typically occurs between 2 and 4 months [9,10,13].

### 2.1. Ethical Approval

This study was conducted in strict adherence to ethical standards, in compliance with French regulations. Ethical approval was granted by the Institutional Review Board (IRB) of the Research, Training and Innovation Directorate of the French Health Service (Direction de la Formation, de la Recherche et de l’Innovation = DFRI), under the approval number 2022HJ17. All procedures performed in the study were in accordance with the ethical standards of the national research committee, and with the 1964 Helsinki declaration and its later amendments, or comparable ethical standards. Additionally, this study was conducted in full compliance with the General Data Protection Regulation (in France, Règlement Général de Protection des Données = RGPD) and adhered to the data protection rules set forth by the French National Commission on Informatics and Liberty (CNIL). These measures ensure the protection of personal data and the privacy of study participants.

### 2.2. Statistical Analysis

A description of the different sociodemographic and clinical variables of the study population was performed. Fisher’s exact test was used to compare qualitative variables, and the Mann–Whitney U test to compare quantitative variables.

Multivariate logistic regression was used to explore factors associated with healing. All variables associated with healing and having a *p*-value < 0.20 in bivariate analyses were included in the initial model, and a backward stepwise variable selection procedure was used to identify factors associated with healing. Variables with a *p*-value > 0.05 were eliminated from the model. The model’s adequacy was tested using the Hosmer–Lemeshow test. All analyses were performed with R software version 4.0.3.

## 3. Results

### 3.1. Patient Characteristics

A total of 160 patients were included in this study. The demographic and clinical characteristics, along with the outcomes of their surgical interventions, are detailed in Table 1. Seven patients were lost to follow-up after the first post-operative consultation. Among the study population, a significant majority (64.4%) were female, reflecting the gender distribution typically observed in HS. The mean age of participants was 32 years. Age distribution varied, with more than half of the patients under 30. Patients’ BMI averaged at 27.1 kg/m^2^, with a substantial portion of the cohort being overweight or obese. A notable 71.2% of the participants were current smokers. Patients were treated with various medical approaches before surgery, with the majority receiving background antibiotherapy (69.9%). The surgical sites varied, with a nearly equal distribution between inguinal/ano-genital and axillary regions. All patients underwent secondary wound healing with daily dressing changes. Additionally, 25 patients (15.6%)—specifically those classified as Hurley stage 3 with axillary lesions and a wound size greater than 50 cm^2^—underwent negative pressure wound therapy (NPWT) for the first three weeks post-surgery, followed by continued daily dressing changes. The mean wound size was 55.2 cm^3^; 95% CI [42.2–67.7].

### 3.2. Analysis According to Healing Time

**Figure 2:**
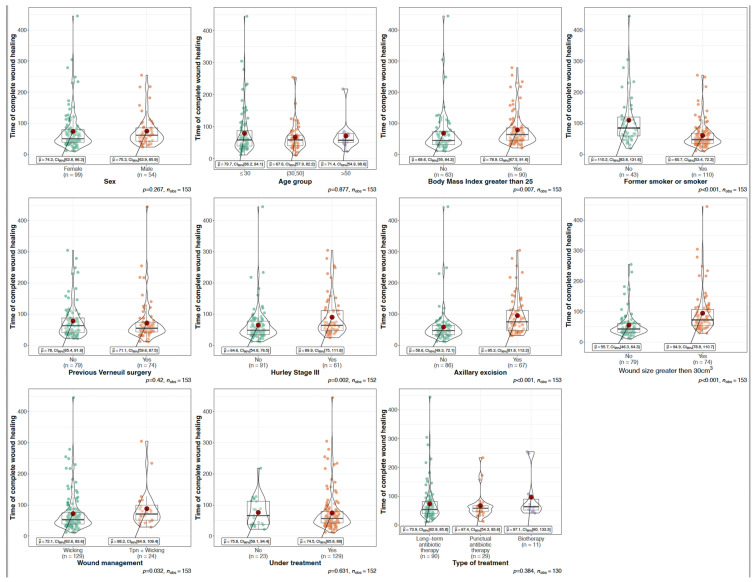
Time to complete wound healing after HS wide excision according to patient characteristics. Legend: **Sex**: Compares the time of complete wound healing between male and female patients. There is no significant difference in healing time between sexes. **Age group**: Healing time across different age groups. Age does not significantly affect healing time. **BMI greater than 25**: Healing time for patients with BMI greater than 25 vs. those with lower BMI; overweight patients (BMI > 25) have a significantly longer healing time. **Former smoker or smoker**: Healing time between smokers and non-smokers. Smokers have a significantly shorter healing time compared to non-smokers. **Previous Verneuil surgery**: Healing time between patients with and without previous Verneuil surgery. Previous Verneuil surgery does not significantly affect healing time. **Hurley Stage III**: Healing time between patients with Hurley Stage III lesions and those without. Hurley Stage III lesions are associated with significantly longer healing time. **Axillary excision**: healing time between patients with and without axillary excision. **Wound size**: Healing time based on wound size greater than 30 cm^3^. Larger wounds (>30 cm^3^) have a significantly longer healing time. **Wound management**: Healing time based on the type of wound management (wound dressing vs. TPN). TPN is associated with a longer healing time compared to wicking alone. **Treatment**: Healing time between patients under treatment and those not under treatment. Ongoing treatment does not significantly affect healing time. **Type of treatment**: Healing time based on the type of treatment (long-term antibiotics, punctual antibiotics, or biologics). The type of treatment does not significantly affect healing time.

The mean time to wound closure (TTWC) was 74.3 days, 95% CI [64.6–84], ranging from 11 to 445 days. Although NPWT followed by dressing showed a higher healing rate (32%) compared to dressing alone (22.2%), the improvement was not statistically significant (*p* = 0.291). Multivariable analysis identified factors associated with increased TTWC: being overweight (BMI > 25 kg/m^2^, *p* = 0.007), Hurley stage III lesions (*p* = 0.002), undergoing axillary excision (*p* < 0.001), wound size >30 cm^3^ (*p* < 0.001) and wound management with NPWT (*p* = 0.032). In contrast, smoking was associated with a reduction in TTWC (*p* < 0.001) (Figure 2). No statistical associations were found with other variables such as sex, age, previous surgery and HS treatments.

### 3.3. Analysis at 90 Days

Patients were analyzed based on their healing status at 90 days post-surgery. Characteristics of patients according to their healing status at 90 days are presented in Table 2. Characteristics influencing healing, analyzed via univariate analysis, showed that current smokers were significantly more likely to have complete healing (86% vs. 52%, *p* < 0.001). Incomplete healing at 90 days was more frequent in patients with inflammatory diseases (*p* = 0.05), axillary HS (*p* < 0.001), Hurley stage III lesions (*p* = 0.017), and larger wound sizes (>30 cm^3^, *p* = 0.004). Ongoing medical treatment for HS was not statistically significant (*p* = 0.057), nor were sex, age, BMI and previous surgery (Table 2).

Multivariate analysis confirmed that current smokers were significantly more likely to achieve complete healing at 90 days compared to non-smokers (interruption of more than one year or never smoker) (OR: 5.18; 95% CI: [2.19–12.82]; *p* < 0.001). Medical treatment, whether antibiotherapy or biologics, also significantly improved healing odds (OR: 4.09; 95% CI: [1.39–12.36]; *p* = 0.011). Conversely, surgeries in the axillary region or in patients with inflammatory diseases were linked to lower healing probabilities. Detailed results are presented in Table 3.

## 4. Discussion

Our study identified several factors significantly influencing the healing process after the wide excision of HS lesions. These findings, especially those related to smoking status, BMI, and wound localization, contribute to a nuanced understanding of post-surgical recovery in HS patients. Wide excision remains the only curative treatment for HS despite advances in biologic therapies, and the better management of surgical outcomes can significantly improve patient care and enhance quality of life.

Surprisingly, current smokers had an almost five times higher chance of healing at 90 days compared to non-smokers (defined as those who never smoked and former smokers who quit more than a year ago). This contrasts with the commonly accepted view that smoking impairs skin and wound healing due to its negative effects on blood flow and immune response [14]. Studies on wound healing usually report a deleterious effect of smoking, leading to delayed healing, linked to the effect of smoking on the tissue microenvironment on inflammatory cellular functions and reparative capabilities [14,15,16,17,18]. These studies were not conducted on HS patients and, indeed, few studies have been carried out on this specific population. For HS, Prens et al. found no differences in healing according to smoking status in a small-sized study of 40 patients [19]. Frings et al. had a similar conclusion [6], but their study population was too small and underpowered to reveal a statistical link between smoking status and the duration of secondary wound healing. While the question of quitting smoking before surgery is always legitimate, our findings suggest that smoking cessation may not be as critical for HS patients undergoing wide excision. Our results suggest that nicotine may play an anti-inflammatory role in patients with HS, thus ruling out the typical negative effects associated with smoking [18,20]. The mode of action is still unknown. An interesting study, published by Jacobi et al. in 2022, showed that nicotine accelerated angiogenesis and wound healing in mice [21]. Since then, few studies have focused on this subject. Given the broader implications of these results, further research is essential to understand the mechanisms through which nicotine influences healing, especially in HS.

Obese individuals are widely reported to have a higher incidence of wound complications, often due to impaired immune response, reduced perfusion, and other obesity-related physiological changes [22,23,24,25]. For HS surgery, Pierpont et al. specifically noted a detrimental impact of obesity on wound healing, which aligns with the broader medical consensus [26]. However, our findings present a nuanced perspective. In our cohort, no significant overall impact of weight on wound healing times was observed after wide excision. Univariate analysis initially suggested that overweight patients (BMI > 25 kg/m^2^) experienced longer healing times (78.9 days compared to 68.6 days for non-overweight patients). This difference was not confirmed in the multivariate analysis, suggesting that when other variables are controlled for, BMI alone may not be as critical a determinant of healing outcomes. Our study’s divergence from these findings highlights the need to consider other factors that may influence wound healing, such as genetic predisposition, levels of local and systemic inflammation, and wound chronicity. Further research is thus warranted to explore the importance of these factors and their interactions in HS specifically, which may lead to more tailored and effective treatment strategies for overweight and obese patients with HS.

Axillary lesions were consistently associated with a longer healing time compared to excisions at other sites. This observation has been described previously [6]. This extended healing duration can be attributed to the unique anatomical and physiological characteristics of the axillary region. Notably, the axillary area has a higher density of pilosebaceous units compared to other common sites of HS involvement, such as the groin and gluteal regions. Additionally, the axillary region exhibits a distinct presence of hair follicle progenitor cells, as indicated by the expression of CK15 and CD200 markers [6]. These biological factors may contribute to more complex wound environments, potentially complicating post-surgical recovery. The complex structure of the axillary region and frequent arm movements complicate healing considerably. This may explain, in part, the often-longer TTWC. It is essential to adapt and refine care plans for each patient, especially for those undergoing multiple surgical procedures. Patients should be well informed that healing times can vary considerably, with the axillary area generally taking longer to heal.

Chronic inflammatory diseases have been linked to non-healing status at 90 days. IBD and other inflammatory diseases have similar clinical manifestations, genetic susceptibility, and immunologic features [27]. IBD and other inflammatory diseases share immunologic features that may exacerbate the inflammatory phase and delay wound healing. While it is known that pro-inflammatory cytokines, such as tumor necrosis factor (TN alpha), prolong the inflammatory phase and delay wound healing [28], more studies are necessary to understand why associated inflammatory diseases slow the healing process after wide excision for HS.

Hurley stage III was associated with a significantly longer healing time compared to Hurley stages I and II (89.9 vs. 64.9 days; *p* = 0.002). This finding is consistent with expectations, as Hurley III represents the most severe and extensive form of the disease, with lesion sizes averaging 71 cm^3^ compared to 44.8 cm^3^ for other stages. The larger lesion size directly contributes to prolonged healing times [29].

Continuing medical treatment, encompassing antibiotics and biologics, demonstrated a positive association with improved wound healing outcomes in our study. However, our analysis did not single out any specific treatment. HS treatment involves both medical and surgical approaches. A comprehensive, multi-modal strategy is essential to manage HS effectively [8,30]. Such proactive management is vital, not only for enhancing immediate post-surgical recovery, but also for preventing the progression of the disease to more severe and debilitating stages. By maintaining therapeutic interventions, especially in the early stages of HS, clinicians may mitigate the severity of the condition, potentially reducing the complexity of the surgical interventions required.

The impact of genetics on HS and its healing process warrants further investigation. One area that requires further study, which was not explored in our research, is the genetic influence on HS. Approximately one third of HS patients have a family history of the condition, suggesting a genetic component [31]. However, the exact role of genetics in HS development is not yet fully understood. Certain genetic mutations, such as those affecting gamma-secretase genes, have been identified in some HS patients [32]. These mutations are thought to impact crucial biological processes, including skin cell growth and differentiation, which could explain variations in disease severity and treatment responses among individuals. A more thorough grasp of the genetic factors involved in HS could facilitate the development of targeted therapies that address the root causes of the disease, improving patient outcomes and quality of life.

NPWT has shown benefits in accelerating wound healing for HS [33]. However, in our study, only 15.6% of patients accepted this device, and its application was limited to three weeks. This approach, including its restriction to post-axillary excision and exclusion from inguinal, gluteal and ano-genital applications due to seal integrity challenges in the folds, may be debatable. The demands of managing NPWT at home, such as device maintenance and discomfort, significantly impacted patients’ quality of life. These challenges underscore the necessity for treatment strategies that effectively balance clinical benefits, practicality, and patient well-being.

Despite the limitations of our retrospective study, including the sample size, the findings provide valuable insights into factors influencing wound healing in HS patients, such as diabetes mellitus and inflammatory diseases. Although our sample size is relatively large compared to previously published studies, we recommend including more patients in future research, ideally through a multicenter study, to confirm and refine our results. A particular limitation in our analysis of smoking’s impact on wound healing was the omission of detailed smoking behavior metrics, such as the number of cigarettes smoked per day and the cumulative exposure measured in pack-years. This oversight may skew the interpretation of nicotine’s influence on healing outcomes, potentially underestimating the variability in smoking habits among our subjects. To address this issue, subsequent studies should include a more detailed assessment of smoking intensity and duration in order to more accurately correlate smoking behavior with healing dynamics in HS. Another point is the absence of preoperative ultrasound mapping in our study protocol. Preoperative ultrasound could significantly enhance surgical planning by identifying deeper abscesses and fistulae, which are common in advanced HS and could affect surgical outcomes, wound healing and relapse.

## 5. Conclusions

This study has underlined several significant factors that influence wound healing in patients undergoing wide excision for HS, offering new insights into the complex interplay of genetics, lifestyle factors and clinical treatments. HS is associated with stress, anxiety and depression, which often lead to smoking. Our study challenges traditional perspectives on smoking and wound healing, suggesting that nicotine may exert a unique anti-inflammatory effect in HS patients. While quitting smoking is generally beneficial, our findings indicate that continuing tobacco use may not substantially impede the healing process in HS surgeries. Axillary lesions, inflammatory disease and ongoing treatments negatively impact healing, whereas overweight and obesity do not. Further research is needed to explore the relationship between smoking and favorable HS surgery outcomes. It is imperative that future research on HS incorporates larger, multicenter studies to validate and expand upon these findings.

## Figures and Tables

**Table 1:** Demographic characteristics of patients who underwent wide excision for hidradenitis suppurativa.

Variable	*n* (%)	95% CI ^a^
**Sex**		
Female	103 (64.4)	[56.6–72.1]
Male	57 (35.6)	[27.9–43.4]
**Age (years), mean (SD ^b^)**	32.5 (11.1)	[30.8–34.3]
**Age group**		
≤30	86 (53.8)	[45.7–61.8]
(30, 50]	59 (36.9)	[29.1–44.7]
>50	15 (9.4)	[4.5–14.2]
**BMI ^c^, mean (SD ^b^)**	27.1 (5.7)	[26.3–28]
**BMI ^c^ group**		
<25	64 (40.0)	[32.1–47.9]
[25, 30)	51 (31.9)	[24.3–39.4]
≥30	45 (28.1)	[20.8–35.4]
**Smoker ^d^**		
No	46 (28.7)	[21.4–36.1]
Yes	114 (71.2)	[63.9–78.6]
**Diabetes mellitus**		
No	153 (95.6)	[92.1–99.1]
Yes	7 (4.4)	[0.9–7.9]
**Inflammatory disease ^e^**		
No	150 (93.8)	[89.7–97.8]
Yes	10 (6.2)	[2.2–10.3]
**Cardiovascular disease**		
No	156 (97.5)	[94.8–100]
Yes	4 (2.5)	[0–5.2]
**Hurley stage III ^f^**		
No	95 (60.1)	[52.2–68.1]
Yes	63 (39.9)	[31.9–47.8]
**ASA Status**		
1	34 (21.2)	[14.6–27.9]
2	120 (75.0)	[68.0–82.0]
3	6 (3.8)	[0.5–7.0]
**Medical treatment**		
No	26 (16.4)	[10.3–22.4]
Yes	133 (83.6)	[77.6–89.7]
**Type of treatment**		
Background antibiotherapy	93 (69.9)	[61.8–78.1]
Antibiotherapy in case of flare-up	29 (21.8)	[14.4–29.2]
Biologics	11 (8.3)	[3.2–13.3]
**Localization excision**		
Inguinal/anogenital	73 (45.6)	[37.6–53.7]
Axillary	70 (43.8)	[35.8–51.7]
Gluteal	15 (9.4)	[4.5–14.2]
Other	2 (1.2)	[0.0–3.3]
**Wound size (cm^3^)**		
≤30	83 (51.9)	[43.8–59.9]
>30	77 (48.1)	[40.1–56.2]
**Time of complete healing > 90 days**		
No	122 (79.7)	[73.0–86.4]
Yes	31 (20.3)	[13.6–27.0]

^a^ Confidence interval. ^b^ Standard deviation. ^c^ Body mass index. ^d^ Smoker = current smoker and former smoker ≤ 1 year. ^e^ Inflammatory disease: inflammatory bowel disease (*n* = 7) or axial spondylarthritis (*n* = 3). ^f^ Three missing values.

**Table 2:** Characteristics of patients according to their healing status at 90 days (wound healing completed or not).

	Time to Complete Healing under 90 Days				
	No		Yes		
	*n* (%)	95% CI ^a^	*n* (%)	95% CI ^a^	*p*
**Sex**					0.858
Female	24 (23.3)	[14.7–32.0]	79 (76.7)	[68.0–85.3]	
Male	14 (24.6)	[12.5–36.6]	43 (75.4)	[63.4–87.5]	
**Age group**					0.854
≤30	22 (25.6)	[15.8–35.4]	64 (74.4)	[64.6–84.2]	
(30, 50]	13 (22.0)	[10.6–33.5]	46 (78.0)	[66.5–89.4]	
>50	3 (20.0)	[0.0–43.6]	12 (80.0)	[56.4–100]	
**BMI ^b^ ≥ 25 kg/m^2^—overweight**					0.404
No	13 (20.3)	[9.7–31.0]	51 (79.7)	[69.0–90.3]	
Yes	25 (26.0)	[16.7–35.3]	71 (74.0)	[64.7–83.3]	
**BMI ^b^ > 30 kg/m^2^—obesity**					0.267
No	30 (26.1)	[17.6–34.5]	85 (73.9)	[65.5–82.4]	
Yes	8 (17.8)	[5.5–30.1]	37 (82.2)	[69.9–94.5]	
**Smoker**					<0.001
No	22 (47.8)	[32.3–63.3]	24 (52.2)	[36.7–67.7]	
Yes	16 (14.0)	[7.2–20.8]	98 (86.0)	[79.2–92.8]	
**Inflammatory disease**					0.058
No	33 (22.0)	[15.0–29.0]	117 (78.0)	[71.0–85.0]	
Yes	5 (50.0)	[14.0–86.0]	5 (50.0)	[14.0–86.0]	
**Localization wide excision**					<0.001
Other	10 (11.1)	[4.1–18.2]	80 (88.9)	[81.8–95.9]	
Axillary	28 (40.0)	[27.8–52.2]	42 (60.0)	[47.8–72.2]	
**Wound size (cm^3^), mean (sd ^c^)**	99.6 (120.3)	[60–139.1]	41.4 (56.4)	[31.3–51.5]	<0.001
**Wound size (cm^3^)**					0.004
≤30	12 (14.5)	[6.3–22.6]	71 (85.5)	[77.4–93.7]	
>30	26 (33.8)	[22.6–45.0]	51 (66.2)	[55–77.4]	
**Post-operative wound management**					0.291
Wound healing by dressing	30 (22.2)	[14.8–29.6]	105 (77.8)	[70.4–85.2]	
NPWT (15 days) following by dressing for wound healing	8 (32)	[11.7–52.3]	17 (68)	[47.7–88.3]	
**Hurley stage III**					0.017
No	16 (16.8)	[8.8–24.9]	79 (83.2)	[75.1–91.2]	
Yes	21 (33.3)	[20.9–45.8]	42 (66.7)	[54.2–79.1]	
**Medical treatment**					0.057
No	10 (38.5)	[17.8–59.1]	16 (61.5)	[40.9–82.2]	
Yes	28 (21.1)	[13.7–28.4]	105 (78.9)	[71.6–86.3]	
**Type of treatment**					0.245
Background antibiotherapy	22 (23.4)	[14.3–32.5]	72 (76.6)	[67.5–85.7]	
Antibiotherapy in case of flare	3 (10.3)	[0–23.2]	26 (89.7)	[76.8–100]	
Biologics	3 (27.3)	[0–58.1]	8 (72.7)	[41.9–100]	

^a^ Confidence interval. ^b^ Body mass index. ^c^ Standard deviation.

**Table 3:** Factors associated with HS healing status at 90 days (wound closed or not) in multivariate analysis.

		Bivariate Analysis			Multivariate Analysis		
		OR	95% CI ^a^	*p*	OR	95% CI ^a^	*p*
**Smoker**	No	-	-	-	-	-	-
	Yes	5.61	[2.59–12.51]	<0.001	5.18	[2.19–12.82]	<0.001
**Inflammatory disease**	No	-	-	-	-	-	-
	Yes	0.28	[0.07–1.07]	0.056	0.17	[0.04–0.73]	0.017
**Localization wide excision**	Other	-	-	-	-	-	-
	Axillary	0.19	[0.08–0.41]	<0.001	0.20	[0.08–0.49]	0.001
**Wound size (cm^3^)**	mean (SD ^b^)	0.99	[0.99–1.00]	0.001	-	-	-
**Hurley stage III**	No	-	-	-	-	-	-
	Yes	0.41	[0.19–0.85]	0.018	-	-	-
**Medical treatment**	No	-	-	-	-	-	-
	Yes	2.34	[0.94–5.68]	0.062	4.09	[1.39–12.36]	0.011

^a^ Confidence interval. ^b^ Standard deviation. Hosmer–Lemeshow goodness-of-fit test *p* = 0.828.

## Data Availability

The data supporting the findings of this study are available upon reasonable request from the corresponding author. Due to privacy and ethical restrictions, the data are not publicly archived. Interested researchers can contact the corresponding author to access the datasets analyzed during the study.
